# Electroacupuncture Preconditioning Alleviates Lipopolysaccharides-Induced Acute Lung Injury by Downregulating LC3‐II/I and Beclin 1 Expression

**DOI:** 10.1155/2022/8997173

**Published:** 2022-10-20

**Authors:** Guanghua Sun, Yahua Zeng, Fu Luo, Lixian Zhang, Jinqu Tan, Jie Tong, Lu Yang, Danni Liu, Liu Liu, Jun Zhou

**Affiliations:** ^1^The First Affiliated Hospital, Department of Rehabilitation, Hengyang Medical School, University of South China, Hengyang 421001, Hunan, China; ^2^The First Affiliated Hospital, Rehabilitation Medicine Center, Hengyang Medical School, University of South China, Hengyang 421001, Hunan, China; ^3^The First Affiliated Hospital, Rehabilitation Laboratory, Hengyang Medical School, University of South China, Hengyang 421001, Hunan, China; ^4^Children's Nerve and Development Center, Maternal and Child Health Hospital of Qingyuan City, Qingyuan 511500, Guangdong, China

## Abstract

Our study aimed to investigate the effect of electroacupuncture pretreatment on the inflammatory response and expression levels of LC3-II/I and Beclin 1 using a model of lipopolysaccharide (LPS)-induced acute lung injury (ALI). Eighteen male Sprague-Dawley (SD) rats were randomly divided into three groups: normal control group (NC, *n* = 6), LSP modeling group (LM, *n* = 6), and electroacupuncture group (EA, *n* = 6). Rats in the EA group received electroacupuncture pretreatment at bilateral Zusanli (ST36) and Chize (LU5) points for five days (30 min each time daily, frequency; 3 Hz/15 Hz, intensity; 1 mA). Rats in the EA and LM groups were then injected with 5 mg/kg LPS (Beijing, Solarbio Company, concentration; 5 mg/mL) through the tail vein, while those in the NC group were injected with 5 mg/kg saline. The animals were sacrificed six hours after LPS or saline injection through cervical vertebrae by dislocation under deep anesthesia. Orbital blood was collected for the analysis of serum inflammatory factors including interleukin-1*β* (IL-1*β*) and transforming growth factor-*β* (TGF-*β*). The lower left lung was excised, stained with hematoxylin-eosin (HE), and subjected to histopathological analysis. The mRNA and protein expression of Beclin 1 and LC3 II/I in the lower right lung tissues were detected via RT-qPCR and Western blot analyses, respectively. The results showed that lung injury score was significantly higher in the LM group than that of the NC group (*P* < 0.01) and EA group (*P* < 0.01). The IL-1*β* contents were significantly decreased in the EA group (*P* < 0.01) than in the LM group. In contrast, the GF-*β* contents were increased in the EA group significantly when compared with the LM group (*P* < 0.01). RT-qPCR and Western blot detection showed that the relative gene expression of LC3‐II/I and Beclin 1 was significantly lower in the EA group than in the LM group (*P* < 0.01). However, the relative protein expression level of LC3‐II/I and Beclin 1 was slightly lower in the EA group than the in LM group (*P* > 0.05). These results show that electroacupuncture pretreatment reduces the inflammatory response in ALI and can protect lung tissue by inhibiting the gene and protein expression levels of LC3‐II/I and Beclin 1.

## 1. Introduction

Acute lung injury (ALI) is caused by various direct and indirect factors that damage alveolar epithelial cells and vascular endothelial cells. It is characterized by pulmonary interstitial, alveolar edema, and respiratory insufficiency. Moreover, severe cases may progress to acute respiratory distress syndrome (ARDS) [1]. It is a common life-threatening lung disease with high mortality and morbidity [2]. ALI is characterized by multiple lung alterations caused by lung injury that was caused by acute and severe lung inflammation. Some ALI cases present with significantly uncontrolled lung inflammation due to activation of inflammatory cells and release of cytokines [3]. As a result, ALI imposes significant economic and social burdens.

Although scientists have made breakthroughs in the development of therapies for ALI, there is no effective treatment for the disease. Besides, the pathogenesis of ALI is currently unclear [4]. Particularly, the Corona Virus Disease 2019 (COVID-19) has ravaged the world and caused severe respiratory distress syndrome [5]. New evidence has shown that COVID-19 survivors may have persistent lung damage [6, 7]. Therefore, it is necessary to explore effective methods for ALI treatment, which may also benefit COVID-19 patients [2].

Previous studies showed that electroacupuncture (EA) pretreatment can significantly reduce the release of inflammatory cytokines and the inflammatory response in rats with lung injury, as well as the secretion of inflammatory factors, such as tumor necrosis factor-*α* (TNF-*α*), interleukin-1 (IL-1), IL-6, and myeloperoxidase [8]. Other studies have demonstrated that EA pretreatment attenuates inflammatory lung injury after cardiopulmonary bypass (CPB) by suppressing NLRP3 inflammasome activation, reducing pulmonary edema, and decreasing the release of inflammatory cytokines into the serum and lungs [9, 10]. EA pretreatment exhibited lung protective and anti-inflammation effects based on the regulation of inflammation factors through SIRT1-related pathways [11].

This study aimed to assess the protective effects of EA pretreatment on lipopolysaccharides (LPS)-induced ALI in rats. The effects of EA on the expression levels of autophagy-related genes and proteins of LC3 and Beclin 1 were also evaluated to clarify the possible theoretical mechanism.

As well known, autophagy is a selfprotection mechanism formed during the evolution of eukaryotic cells [12]. The autophagy-related gene Beclin 1 is involved in major autophagy processes, and it is essential to the formation of autophagosomes and an important regulator in the autophagy pathway [13, 14]. The LC3‐II and Beclin 1 proteins are markers of autophagy [15]. Research suggests that when autophagy fails, cellular, tissue, or organismal manifestations often present as dysregulated inflammation and other abnormalities [16]. However, the regulation of autophagy during the development of ALI is not well understood [17]. Studies have shown that low, insufficient, or excessive autophagy in lung tissue can cause or aggravate lung injury [18]. The production of inflammatory cytokines in LPS-induced ALI can be inhibited by the autophagy-related PI3K/AKT/mTOR pathway, and the autophagy-related proteins (LC3-II and Beclin 1) were upregulated in lung tissue after 6 hours of intratracheal treatment with LPS [19]. Therefore, studying autophagy status may reveal ideas for the prevention and treatment of ALI by electroacupuncture.

## 2. Materials and Methods

### 2.1. Animals

Eighteen three-month-old clean‐grade Sprague‐Dawley male rats (370-487 g) were purchased from Changsha Tianqin Biological Technology Co Ltd, Hunan Province (certificate number: SCXK (Xiang) 2019-0004). The animals were kept in the Department of Experimental Animal Laboratory, University of South China, Hengyang, Hunan Province (room temperature: 24 ± 2°C, humidity: 55 ± 5%, and 12-hour dark/light cycle). The rats were given free access to water and food. The follow-up experiment was conducted after one week of adaptive feeding.

### 2.2. Equipment

Fluorescence quantitative PCR instrument (PikoReal 96) and fluorescence PCR plate (SPL0960) were obtained from Thermo (Thermo, USA). Decoloring shaker (TS-1) and vortex mixer (GL-88B) were bought from Qilinbeier (Jiangsu, China). Tabletop refrigerated centrifuge (H1650R) was obtained from Xiangyi (Hunan, China). Membrane transfer apparatus, electrophoresis apparatus, and horizontal agarose electrophoresis tank were purchased from Liuyi (Beijing Liuyi Biological Technology Co Ltd, China). Magnetic stirrers (JB‐13) were purchased from INESA scientific instrument (Shanghai, China). An optical microscope was obtained from Olympus Corporation, Japan. The slicing machine was purchased from LEICA (Germany). A multifunctional enzyme marker analyzer was obtained from Heales (Shenzhen, China). SDZ‐V nerve and muscle stimulator were purchased from Hwato (Suzhou Medical Appliance Factory, China). Primer synthesis and probe modification were conducted in Sangon Biotech (Shanghai, China).

### 2.3. Reagents

Lipopolysaccharides were purchased from Solarbio (Beijing Solarbio, China). Interleukin‐1*β* (IL‐1*β*) and transforming growth factor‐*β* (TGF‐*β*) ELISA kits were obtained from Thermo Fisher Scientific (USA). Agarose was sourced from BIOWEST (BIOWEST, Spain). EDTA and SDS were obtained from Meilunbio (Dalian, China). The mRNA and miRNA reverse transcription kits were purchased from CoWin Biosciences (CWBIO, China). Tris, DEPC, glycine, methylene bisacrylamide, ultra SYBR Mixture, and DM2000 Plus DNA Marker were purchased from Sigma, USA. TRIzol was obtained from Thermo, USA. APS, Tween‐20, and Ponceau dye were sourced from SINOPHARM (Shanghai, China). TEMED was sourced from Aladdin (Shanghai, China). Acrylamide and protease inhibitors were purchased from Gentihold (Beijing, China). BSA was obtained from Yancheng Saibao (China). RIPA lysis buffer was purchased from Beyotime Biotechnology, China. SuperECL Plus Western Blotting Substrate was purchased from Advansta, USA. Anti-Beclin 1 (ab62557, 1 *μ*g/ml) was obtained from Abcam, Britain. Anti-LC3 was obtained from CST, USA., while antiactin was obtained from Proteintech, USA.

### 2.4. Establishment of ALI Animal Model

The experimental operation room was sterilized using ultraviolet light for 15 min. An LPS solution with a concentration of 5 mg/ml was prepared for the experiments. The rats were weighed and placed in a fixed cylinder while their tails were outside the cylinder. The surface of the rat tail vein was disinfected using a 75% alcohol cotton ball. Using a previously reported method, the skin of the tail vein (filled) was pierced with a 1 ml syringe at the lower 1/3 of the tail vein at 45 degree [20]. The direction of the injection needle was then adjusted at the level of the blood vessel. A visible small amount of dark red blood confirmed that the needle tube was in the blood vessel. The rats were injected with the prepared LPS solution (5 mg/kg per body weight) to establish an ALI rat model. All the rats were returned to the cage after confirming that they were normal.

### 2.5. Intervention Methods

The rats were fed on a normal diet during the intervention period after one week (adaptive feeding). The rats were randomly divided into three groups using a random number table generated by SPSS software: the normal control group (NC, *n* = 6), LSP model group (LM, *n* = 6), and electroacupuncture group (EA, *n* = 6). The body weight of the rats was not significantly different among the groups (*P* > 0.05).

The rats in the EA group were subjected to acupuncture at Chize (LU5) and Zusanli (ST36) for five days before LPS injection. Traditional acupuncture points were obtained from the “Map of the Experimental Animal Acupuncture Points,” developed by the Experimental Acupuncture Institute of China Association of Acupuncture and Moxibustion.

Disposable sterile steel acupuncture needles (diameter: 0.25 mm and length: 25 mm) (Huatuo, Suzhou Medical Instruments Factory, China) were used in this study. The acupuncture needles were perpendicularly inserted at LU5 (depth: 2-3 mm) and ST36 (depth: 3-5 mm). Bilateral pairs were interconnected to the Hwato SDZ‐V acupoint stimulator EA apparatus (density wave, frequency 3 Hz and 15 Hz, intensity: 1 mA). The LPS (5 mg/kg) was injected on day 5 through the tail vein after EA. The rats were sacrificed 6 hours after LPS injection.

Rats in the LM group were injected with LPS solution only into the tail vein on the 5th day, whereas rats in the NC group received a similar dose of saline.

### 2.6. Specimen Collection

Six hours after LPS injection, all rats were anesthetized with an intraperitoneal injection of pentobarbital (50 mg/kg). Orbital blood (4 mL) was obtained before the rats were sacrificed via cervical dislocation. The blood was stored at room temperature for 2 h. It was subsequently centrifuged (4°C, 2000 × *g*) for 15 min. The collected supernatant was stored at −80°C for the ELISA test.

The lower left lung tissues were fixed in paraformaldehyde solution (4% PFA) for pathological analysis. The lower right lung tissues were put into a cryopreservation tube and quickly transferred to a liquid nitrogen tank for cryopreservation. RT-PCR and Western blot tests were performed to determine expression levels of target genes and proteins.

### 2.7. Determination of Serum IL-1*β* and TGF-*β* Contents

ELISA kit was used to detect the content of IL‐1*β* and TGF‐*β* in the blood supernatant stored in a −80°C refrigerator.

### 2.8. Lung Tissue Hematoxylin-Eosin Staining and Lung Histopathology Semiquantitative Score

The left lower lung tissues fixed in the paraformaldehyde solution were dehydrated and treated with xylene to make them transparent. The specimen was then immersed in melted paraffin, embedded in paraffin, sliced, and attached to a glass slide for HE staining. The specimens were dewaxed, washed using distilled water, stained with HE and ammonia water, dehydrated with ethanol, and sealed with neutral gum.

An optical microscope was used to observe the pathological changes of lung tissues in each group. A semiquantitative scoring system was used to evaluate the lung injury features (refer to standard) [21], including alveolar hyperemia, hemorrhage, neutrophil infiltration or aggregation in the air cavity or vascular wall, alveolar wall thickness transparent membrane formation, and inflammatory cell infiltration. Lung injury scores (0-16) were classified as follows: 0 represents no injury or very slight injury, 1 represents mild injury, 2 represents moderate injury, 3 represents severe injury, and 4 represents very severe injury.

### 2.9. Measurement of mRNA Relative Expression Levels of Beclin 1 and LC3 B

TRIzol reagent was used to extract total RNA from about 0.02 g of the right lower lung tissue stored in the liquid nitrogen tank. The cDNA was reverse transcribed using the total tissue mRNA as a template. The sequence of the target gene was obtained from the NCBI. The target gene primers were designed using Primer 5 software. RT-qPCR was performed using cDNA as a template, and the relative mRNA expression level of each gene was analyzed in reference to *β*-actin. The primer sequences are shown in Table 1. The number of amplification cycles (Ct value) was measured using the PCR instrument, and relative mRNA levels of Beclin 1 and LC3 B were calculated using the 2^−△△Ct^ method.

### 2.10. Protein Expression Levels of Beclin 1, LC3‐I, and LC3‐II

About 0.025 g of the lower right lung tissue was cut and rinsed with ice precooling PBS solution. The tissue was repeatedly grounded using a biological sample homogenizer after lysis for 10 min. The sample was then centrifuged (4°C, 2000 rpm) for 15 min, and the supernatant was transferred to a centrifuge tube. The following procedure was used to prepare a gel: electrophoresis (constant voltage: 75 V, time: 130 min), gel cutting (Beclin 1 (52 KD), LC3 (14,16 KD), and actin (42 KD)), and film transferring (300 mA constant current). Beclin 1 was about 72 min, LC3 was about 30 min, and actin was about 62 min. The sample was blocked and incubated with primary (Beclin 1 and LC3) and secondary antibodies. The ECL color development reagent was added and the membrane was exposed. The exposed film was scanned, and its quantity was analyzed using professional grayscale analysis software.

### 2.11. Statistical Analysis

This was a completely randomized design experiment. The data were processed using the SPSS 22.0 software. The normal distribution of measurement data of each group was tested. The data with normal distribution were expressed as mean ± SD. One‐way analysis of variance (ANOVA) and Dunnett's test were used to compare differences among groups. Non‐normally distributed data were expressed as median and quartile [M (P25, P75)] using the rank-sum test and Kruskal–Wallis 1‐way ANOVA (K samples) test. *P* < 0.05 was considered as a statistically significant difference. GraphPad Prism 9.0 software was used to prepare statistical graphs.

## 3. Results

### 3.1. Serum IL-1*β* and TGF-*β* Contents

**Figure 1:**
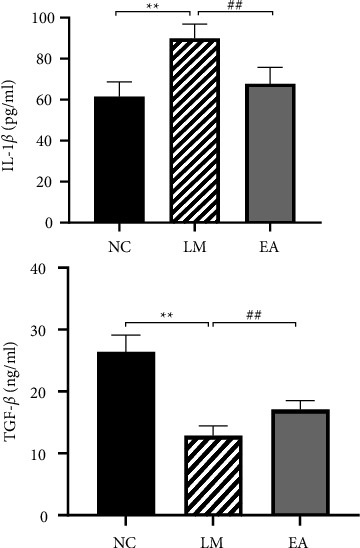
Serum IL-1*β* and TGF-*β* contents. Data were presented as mean ± SD, *n* = 6.  ^*∗∗*^*P* < 0.01 versus NC group. ^##^*P* < 0.01 versus LM group.

The content of serum IL-1*β* and TGF-*β* in all groups is shown in Figure 1. Compared to the NC group, the level of IL-1*β* was significantly higher in the LM group (*P* < 0.01). In contrast, TGF-*β* in the LM group was significantly lower than that in the NC group (*P* < 0.01). The degree of LPS-induced lung injury was lower in the EA pretreated group than that in the LM group. Further analysis showed that IL-1*β* content was significantly lower in the EA group (*P* < 0.01), while the TGF-*β* content was significantly higher (*P* < 0.01) following EA treatment.

### 3.2. Histopathological Semiquantitative Evaluation of Acute Lung Injury

**Figure 2:**
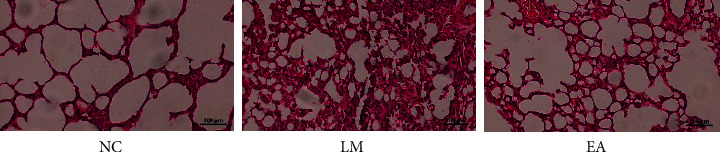
Comparison and analysis of pathological sections of lung tissues of rats in each group (HE staining ×200 times).

The lung condition of rats in all groups was observed using the HE staining assay (Figure 2). The alveolar structure of the NC group was normal, the lung tissue structure was clear, and the alveolar wall was intact. No significant congestion and hemorrhage, neutrophil aggregation, or hyaline membrane formation occurred in the NC group. The alveolar wall of the LM group was thickened, partially destroyed, and fused and had significant congestion after LPS injection compared with the NC group. Significant inflammatory cell infiltration occurred in the lung interstitium of the LM group. Moreover, red blood cell exudation occurred in the alveoli of the LM group. Lung injury of rats in the EA group was significantly reduced compared with the LM group. However, partial thickening and destruction of alveolar walls and the aggregation of neutrophils occurred in the EA group.

**Figure 3:**
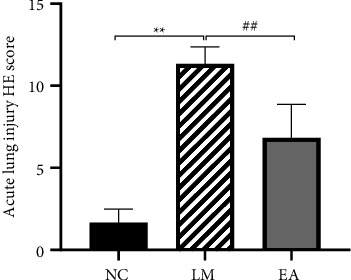
Histopathological semiquantitative evaluation of acute lung injury. Data were presented as mean ± SD. *n* = 6.  ^*∗∗*^*P* < 0.01 versus NC group. ^##^*P* < 0.01 versus LM group.

The semiquantitative score of lung histopathology is shown in Figure 3. The lung injury score of the LM group was higher than that of the NC group (*P* < 0.01). In comparison, the score was higher in rats treated with EA than rats in the NC group (*P* < 0.01).

### 3.3. mRNA Relative Expression Levels of Beclin 1 and LC3B

**Figure 4:**
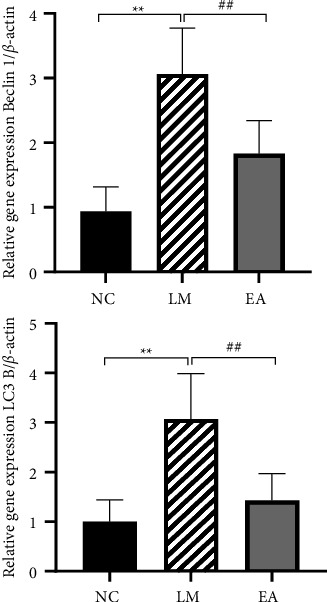
mRNA relative expression levels of Beclin 1 and LC3B. Data were presented as mean ± SD.  ^*∗∗*^*P* < 0.01 compared with the NC group, ^##^*P* < 0.01 compared with the LM group.

Results shown in Figure 4 show that LC3 B and Beclin 1 expression in the lung tissue was significantly lower in the EA group than in LM and NC groups (both *P* < 0.01). LPS increased the mRNA relative expression levels of Beclin 1 and LC3 B, whereas EA pretreatment reduced the expression levels of these indicators.

### 3.4. Protein Expression Levels of Beclin 1, LC3‐I, and LC3‐II

**Figure 5:**
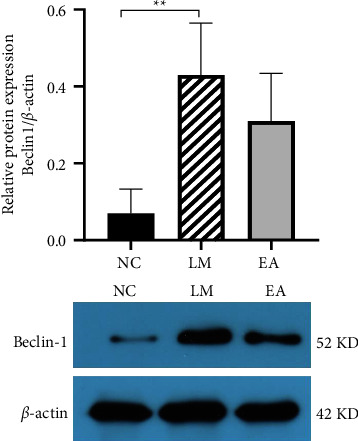
Protein expression levels of Beclin 1. Data were presented as mean ± SD. ^*∗∗*^*P* < 0.01 compared with the NC group.

Figure 5 shows the protein levels of Beclin 1, LC3‐I, and LC3‐II in lung tissues. Notably, the relative expression level of Beclin 1 in the LM group was significantly higher than the NC group (*P* < 0.01). Moreover, the expression level of Beclin 1 was moderately lower in the EA group than the LM group (*P* > 0.05). EA pretreatment reduced the expression of Beclin 1 in the LPS-induced-ALI rats.

**Figure 6:**
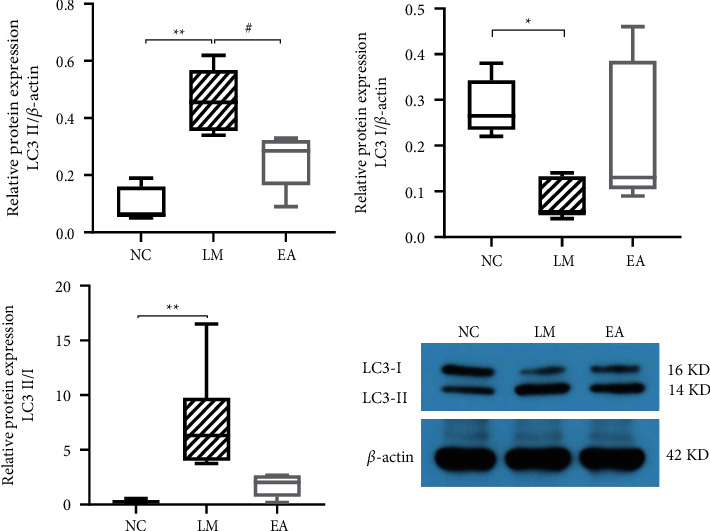
Protein expression levels of LC3-I and LC3-II. Data were presented as median and quartile [M (P25, P75)].  ^*∗∗*^*P* < 0.01 compared with the NC group,  ^*∗*^*P* < 0.05 compared with the NC group. ^#^*P* < 0.05 compared with the LM group.

Besides, the relative protein expression of LC3-II was increased in the LM group significantly than in the NC group (*P* < 0.01). In contrast, the expression of LC3-I of the LM group was remarkably reduced in the LM group versus the NC group (*P* < 0.05). Furthermore, the protein expression level of LC3-II was significantly lower in the EA group than in the LM group (*P* < 0.05). In contrast, the protein expression level of LC3-I was slightly higher in the EA group than in the LM group (*P* > 0.05) (Figure 6). Nevertheless, LC3-II/I expression was significantly higher in the LM group than in the NC group (*P* < 0.01). Moreover, EA pretreatment slightly inhibited LC3-II/I levels (*P* > 0.05).

## 4. Discussion

This study found the positive effects of EA pretreatment in the prevention and treatment of ALI. For instance, EA pretreatment improved LPS-induced ALI inflammatory response by decreasing the content of IL‐1*β* and increasing TGF‐*β* content. EA pretreatment also downregulated the gene and protein expression levels of Beclin 1 and LC3. These results show that EA pretreatment can effectively reduce ALI severity and enhance lung function by inhibiting the mRNA and protein expression levels of autophagy-related genes (Beclin 1 and LC3-II/I) in lung tissue.

Acupuncture and moxibustion have been used to treat lung inflammation for several years. Acupuncture is a safe and effective green therapy that has been gradually accepted and recognized internationally. The China Association of Acupuncture-Moxibustion issued the first and second editions of guidance for acupuncture and moxibustion interventions on COVID-19 in February and March 2020, respectively [22]. In acupuncture and moxibustion therapy, acupoints and compatibility are determined based on the syndrome differentiation and treatment. The therapy can stimulate righteous qi, thus tonifying qi and strengthening exterior, clearing lungs and removing phlegm, nourishing the lung and kidney, heat-clearing (Qingre), and detoxifying (Jiedu). Moreover, acupuncture has been used to treat various pulmonary diseases [23–25].

It was recorded as early as the Inner Canon of Yellow Emperor that “the sages usually focus on prevention instead of the treatment of a disease or regulating a disorder (superior prevention of illness).” As a result, EA pretreatment has been used to prevent and treat diseases of multiple systems, including the respiratory, cardiovascular, and cerebrovascular systems [26]. In this study, we found that EA pretreatment ameliorated ALI and effectively reduced its severity of ALI.

Some studies have suggested that EA can reverse pulmonary inflammation, oxidative damage, and apoptosis by reducing phosphorylation of p38, activating caspase-3 [27], activating the nuclear factor erythroid-2-related factor-2/heme oxygenase (Nrf2/HO-1) pathway through p38 MAPK [28], inhibiting ROS/Nrf2/NLRP3 pathway [29], and activating NLRP3 inflammasomes [9]. These approaches can reduce pulmonary edema, and hence inhibit inflammation-induced lung damage. Our results also showed that EA pretreatment improved LPS-induced ALI inflammatory response by decreasing the content of IL‐1*β* and increasing TGF‐*β* content.

Herein, two acupoints, Zusanli (ST36) and Chize (LU5), were selected for EA pretreatment to assess the therapeutic effect of EA based on autophagy. Zusanli (ST36) is the he-sea point for the combination of the stomach meridian and the main point for health care. It can regulate the spleen and stomach, strengthening the body and vitality. Modern research has shown that Zusanli can also regulate the body's immunity, enhancing disease resistance [30]. Chize (LU5) is the he-sea point of the main meridian of the lung meridian and is the acupuncture point for respiratory diseases. EA at Zusanli (ST36) and Chize (LU5) can improve lung function and morphology [31], thus significantly increasing the oxygenation index of lung injury, probably by decreasing TNF‐*α* content or increasing IL‐10 content [32]. Acupuncture at ST36 point is a promising, supplementary method for controlling sepsis inflammation, thus reducing the damage to the heart, lung, kidney, liver, gastrointestinal tract, and immune system [33] probably by reducing oxidative stress and inflammation [34], improving microcirculation disorders, maintaining dopamine-mediated immune balance [33], and inhibiting toll‐like receptor 4 (TLR4)/NF‐*κ*B pathway [8] to reduce the release of inflammatory factors effectively. Herein, these effects were weakened in the EA group [35, 36]. Consistent with findings from previous studies, our results show that EA can effectively prevent LPS-induced ALI inflammatory response.

Autophagy is a selfprotection mechanism formed by eukaryotic cells during evolution. Moreover, a chronic mild stress response in cells involves transporting damaged proteins to lysosomes for degradation and circulation to maintain cell structure, function, and metabolic stability [37]. The autophagy-related gene, Beclin 1, is involved in major autophagy processes essential for forming autophagosomes, and it is an important regulator of the autophagy pathway [13]. Beclin 1 has also been shown to initiate autophagy. LC3-II can be used as a marker for autophagosome formation [14]. The ratio of LC3-II/I can estimate autophagy levels. This study found that EA pretreatment downregulated the expression levels of Beclin 1 and LC3-II/I genes and proteins in ALI model rats. To a certain extent, we speculate that EA pretreatment may reduce autophagy levels in lung tissue. However, this hypothesis needs to be investigated using autophagy inhibitors or observation of tissue autophagosome formation under a transmission electron microscope.

Recent studies have confirmed that autophagy dysfunction can lead to increased inflammation and oxidative stress, alveolar apoptosis, cell senescence, and repeated infections, thus promoting acute or chronic respiratory diseases [38–43]. Autophagy plays a protective role by regulating inflammation-oxidative stress, apoptosis, and pathogen removal mechanisms [44]. Moreover, LPS-induced lung inflammation weakens the immunosuppressive effect of regulatory T cells (Tregs), which maintain immune homeostasis through autophagy [45]. Therefore, autophagy inhibition can reduce LPS-induced lung inflammation [46] and the severity of ALI [47] by reducing the lung weight factor in bronchoalveolar fluid (BALF) and TNF-*α*, or increasing PaO2 content [48]. Moreover, the inhibition of endoplasmic reticulum stress and autophagy through the classic AKT/mTOR signaling pathway can prevent the activation of the NF-*κ*B pathway and reduce the release of LPS-induced proinflammatory mediators such as IL-1*β*, TNF-*α*, and IL-6 [49]. Neutrophil autophagy inhibition has a protective effect on LPS-induced ALI [50]. Autophagy inhibition can also improve ALI in diabetic rats [51]. However, autophagy inhibition following Beclin 1 knockdown can cause various types of cancers (such as lymphoma, liver cancer, and lung cancer) [52]. Another study inhibition can exacerbate ALI induced by copper oxide nanoparticles (CuONPs) [53]. Studies have shown that regulated autophagy is a survival mechanism that protects cells from starvation, hypoxia, and infection, while overactivated autophagy can lead to cell apoptosis or necrosis [54]. Therefore, maintaining a moderate level of autophagy can reduce lung damage and promote cell survival in the body. Unfortunately, the research group did not explore the signaling pathways targeted by EA preconditioning in ALI. In the future, we will investigate the mechanism using well-designed studies with large samples.

It has been shown that EA can affect pulmonary inflammation by regulating autophagy. For instance, high autophagy levels in rats with COPD aggravated airway inflammatory responses and decreased autophagy levels produced opposite effects [55]. Moreover, EA at Zusanli (ST36) and Feishu (BL13) can inhibit autophagy in lung tissue in rats with COPD, decrease the expression level of Beclin 1 and the ratio of LC3-II/I, and reduce lung inflammation to improve lung function [56]. Similarly, we found that EA inhibited gene and protein expression levels of Beclin 1 and LC3-II/I in lung tissue to ameliorate ALI. Together, these findings strongly support EA as a potential therapy for ALI.

## 5. Conclusions

This study shows that EA prevents ALI by downregulating Beclin 1 and LC3‐II/I expression levels. The ongoing COVID-19 pandemic has been a serious challenge worldwide. This study indicates that traditional Chinese medicine can be used for the prevention and treatment of COVID-19 and provides a strong rationale for the adoption of acupuncture in the treatment of ALI.

## Figures and Tables

**Table 1:** Primer sequences and product lengths.

Gene	Sequence	Product length
*β*‐actin	F ACATCCGTAAAGACCTCTATGCC	223 bp
	R TACTCCTGCTTGCTGATCCAC	

Beclin 1	F GTGGCGGCTCCTATTCCATC	106 bp
	R GACACCCAAGCAAGACCCCA	

LC3 B	F AACACAGCCACCTCTCGACCT	125 bp
	R ACACAACCCACACACGGCAG	

## Data Availability

Data are available on request.

## Abbreviations

ALI: Acute lung injury
SD: Sprague-Dawley
EA: Electroacupuncture
ARDS: Acute respiratory distress syndrome
COVID-19: Corona Virus Disease 2019
LPS: Lipopolysaccharides
IL-1*β*: Interleukin-1*β*
TGF-*β*: Transforming growth factor-*β*
TNF-*α*: Tumor necrosis factor-*α*
CPB: Cardiopulmonary bypass
CuONPs: Copper oxide nanoparticles.
